# Quantitative three-dimensional image analysis of the superior canal after surgical plugging to treat superior semicircular canal dehiscence

**DOI:** 10.1038/s41598-021-95063-x

**Published:** 2021-08-09

**Authors:** Sang-Yeon Lee, Yein Lee, Jun Young Choi, Yun Jung Bae, MinJu Kim, Jae-Jin Song, Byung Yoon Choi, Won-Ki Jeong, Ja-Won Koo

**Affiliations:** 1 Department of Otorhinolaryngology-Head and Neck Surgery, Seoul National University Hospital Seoul National University College of Medicine, Seoul, South Korea; 2 Department of Computer Science and Engineering, Korea University, Seoul, South Korea; 3 Department of Computer Science and Engineering, Ulsan National Institute of Science and Technology (UNIST), Ulsan, South Korea; 4 Department of Radiology, Seoul National University Bundang Hospital, Seongnam, South Korea; 5 Department of Otorhinolaryngology-Head and Neck Surgery, Seoul National University Bundang Hospital, Seongnam, South Korea

**Keywords:** Diseases, Medical research, Signs and symptoms

## Abstract

Surgical plugging to treat superior semicircular canal dehiscence (SCD) has been proven to impede the effect of the third mobile window, abating cochleovestibular symptoms. Knowledge of superior semicircular canal (SC)-plugging status has been proposed to serve as a guide for adjuvant treatment. Here, we investigated disturbances in the inner ear fluid space following SC plugging using a novel three-dimensional (3D) reconstruction-based method. This approach used a semi-automatic segmentation algorithm and a direct volume rendering method derived from conventional magnetic resonance images. The variable extents of filling defects at the sites of SC plugging and the positional relation of the defect to the ampulla and common crus were identified. The success group exhibited markedly reduced volumes following surgery, whereas the failure group displayed no changes in volume. These results indicate that the success or failure of SC plugging was related to 3D volume changes in the labyrinth fluid signal. Collectively, this study presents individualized SC-plugging statuses using a novel 3D reconstruction-based method and it facilitates future work regarding easy-to-measure 3D volume changes. This current technology also aids in the exploration of pathologic changes in various targets of interest.

## Introduction

Superior semicircular canal dehiscence (SCD) syndrome is characterized by the presence of a third mobile window, which can cause clinically debilitating cochleovestibular symptoms such as autophony, tinnitus, sound-induced vertigo (i.e., Tullio phenomenon), pressure-induced nystagmus (i.e., Hennenbert sign), and dizziness, due to a bony defect in the superior semicircular canal (SC)^1–4^. The site of dehiscence or thinning of the SC is most often the arcuate eminence, where the most superior part of the SC faces the middle cranial fossa. Dehiscence or thinning of the SC occasionally occurs at the superior petrosal sinus (SPS), allowing the pressure- or sound-induced motion of the cochlear fluid to shunt into the vestibular organ^1^. Hypermobile fluid dynamics in the otic capsule presumably constitute an important mechanism that increases the pressure shunt into the vestibular end organ^5^. Surgical plugging of a dehiscent SC can impede the effect of the third mobile window, thus immediately reducing subjective symptoms and restoring cochleovestibular hyper-responsiveness^2^.

Imaging plays a crucial role in the diagnosis of SCD. Coronal reformations from high-resolution computed tomography (CT) images of the temporal bone are essential for the diagnosis of SCD^6^. Moreover, magnetic resonance imaging (MRI) using fast imaging with a steady-state acquisition sequence can reliably exclude a SCD diagnosis, with a negative predictive value of 100%^7^. High-resolution MRI reformatted to the plane of the SC, such as a three-dimensional (3D) multiplanar and/or maximum intensity projection reconstruction, can help with visualizing the signal from residual fluid in the SC following canal plugging^2^. Based on 3D T2-weighted images, a normal status after SC plugging is reportedly achieved in fewer than 70% of affected patients, presumably due to a filling defect involving the superior ampulla or incomplete plugging^8^. Information is needed concerning the occurrence of incomplete plugging or the displacement of plugging materials that may cause lasting subjective symptoms. This information would provide additional basis for adjuvant treatment strategies and may permit volumetric evaluation of the SC-plugging state.

There is increasing evidence that preoperative quantification of SC dehiscence permits comprehension of the pathophysiological mechanism underlying the third mobile window, as demonstrated by correlations of the preoperative extent of dehiscence with subjective and objective measures of cochleovestibular functions^9–11^. However, techniques to measure dehiscence size vary among studies, resulting in controversy concerning the use of dehiscence quantification in clinical assessment. Furthermore, two-dimensional length or width calculations largely utilized thus far may lead to biased results^12, 13^, due to the small size and complex pathology of the labyrinth. Two-dimensional reconstruction alone cannot be used to estimate the status of a filling defect or the extent of plugging involving the ampulla. Thus, a de facto 3D reconstruction technique is needed.

We recently reported that the plugging of a dehiscent SC resolved diverse symptoms caused by the third mobile window, whereas the vestibulo-ocular reflex (VOR) gain in the SC was not significantly attenuated. Notably, the gain recovered over time from the initial attenuation observed immediately after surgery^2^. A whole semicircular canal may therefore be unnecessary for the production of appropriate VOR. The desired VOR might be achieved by plugging limited to the dehiscent site (i.e., an ampulla-sparing approach). We aimed to determine the extent of inner ear fluid space disturbance due to surgical plugging, by means of MRI evaluation. Furthermore, volumetric changes between preoperative and postoperative statuses have not been investigated using a 3D reconstruction modality, which can better represent the complex morphometry of the plugging state. This modality may also confer better visualization and allow meticulous quantification of the pathologic lesion.

Herein, we describe a novel 3D measurement method that combines manual segmentation of the labyrinth and an automatic thresholding algorithm to visualize and quantify the surgical plugging status of a dehiscent SC. This work supports future investigations regarding 3D-based easy-to-measure assessments of the status and extent of SC plugging based on conventional MRI, which are accessible in all institutions. We also aimed to obtain preliminary data from pre- and post-operative comparisons of 3D reconstruction-based dehiscence volume, which was correlated with clinical parameters.

## Materials and methods

### Patients

**Figure 1:**
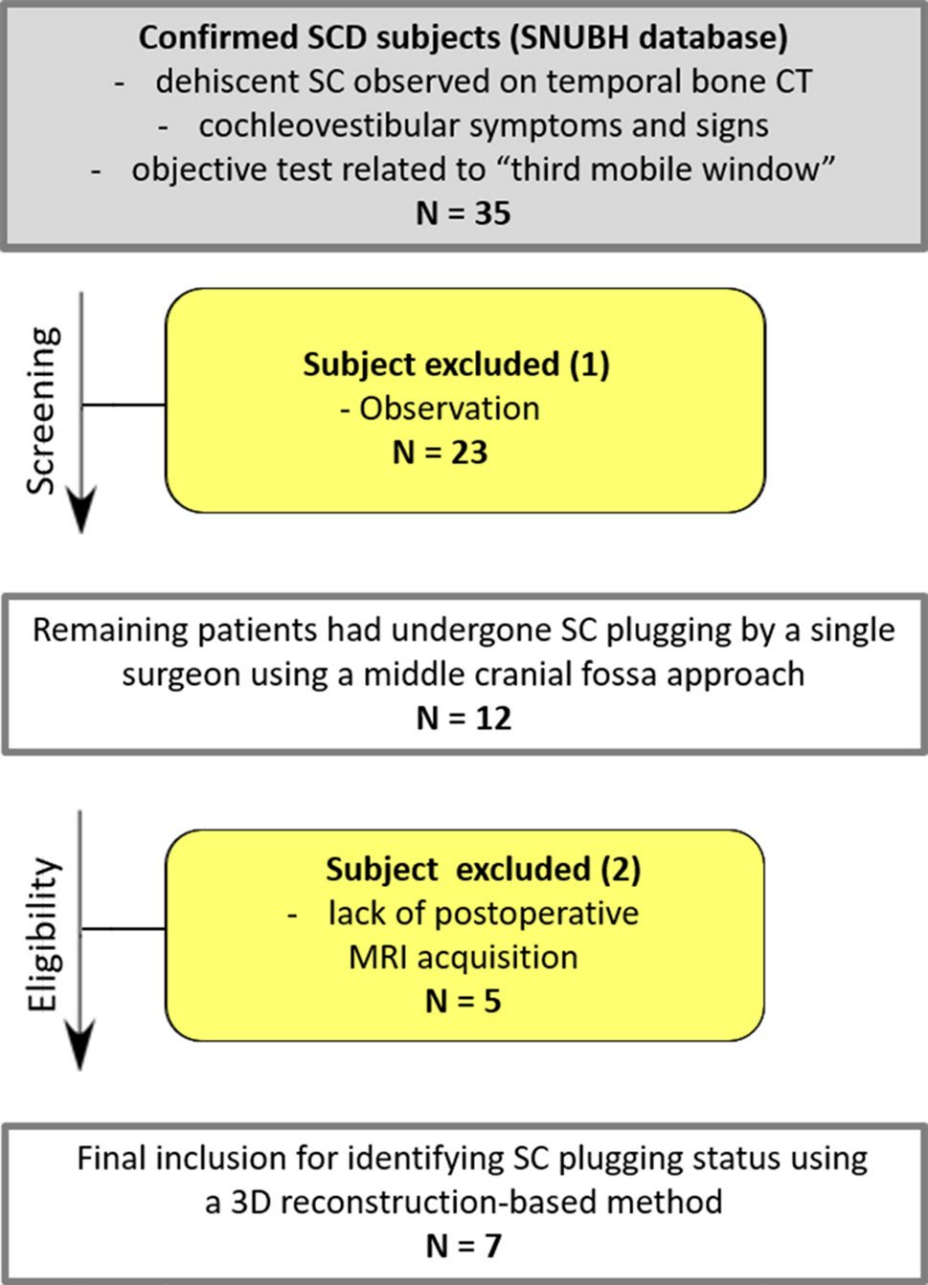
Flow diagram of the inclusion and exclusion criteria for identifying superior semicircular canal (SC)-plugging status using a 3D reconstruction-based method. *SCD* superior semicircular canal dehiscence, *CT* computed tomography, *3D* three-dimensional, *SC* superior semicircular canal.

This retrospective study initially included 35 patients diagnosed with SCD at Seoul National University Bundang Hospital between January 2017 and December 2019 (Fig. 1). Patients with other otologic disorders, such as Meniere’s disease and otosclerosis, were not included. The diagnostic criteria for SCD included dehiscent SC observed on high-resolution temporal bone CT reformatted in the plane of the SC, cochleovestibular symptoms and signs, and objective analysis of abnormal pressure transmission through a third mobile window. Of the 35 patients, 23 who wanted ‘observation’ were excluded. The 12 remaining patients had undergone SC plugging by a single surgeon (J.W.K.) using a middle cranial fossa approach. Further, five were excluded due to a lack of postoperative MRI data. Thus, seven patients were included in the final analysis. The study protocol and a waiver of consent for this retrospective chart review were approved by the review board of the Clinical Research Institute at Seoul National Bundang Hospital (approval no. B-2004-604-125). All methods employed in this study were in accordance with the approved guidelines and the Declaration of Helsinki.

### Surgical intervention

Canal plugging was performed to occlude canal defects without manipulating those defects. Using a middle cranial fossa approach, a combination of soft tissue and bone wax was used to ensure a watertight seal, which was then covered with bone chip and periosteum to tighten the occlusion for the dehiscent SC^2^.

### Imaging

MRI examinations were performed using a 3 T magnetic resonance scanner (Ingenia; Philips Healthcare, Amsterdam, Netherlands) with a 32-channel SENSE Head Coil (Philips Healthcare). Heavily T2-weighted images were taken using 3D T2-weighted volume isotropic turbo spin-echo acquisition. The imaging parameters were as follows: repetition time, 2000 ms; echo time, 258 ms; flip angle, 90°; echo train length, 74; number of excitations, 1; slice thickness, 0.35 mm; and overlap, 0.35 mm. Due to differences in field of view and acquisition matrix between individuals, we have resized size (voxel) and image resolution per voxel (*mm*3 per voxel) (Supplementary Table 1). In this study, MRI was performed over a 3-month follow-up period after SC plugging in all patients.

**Figure 2:**
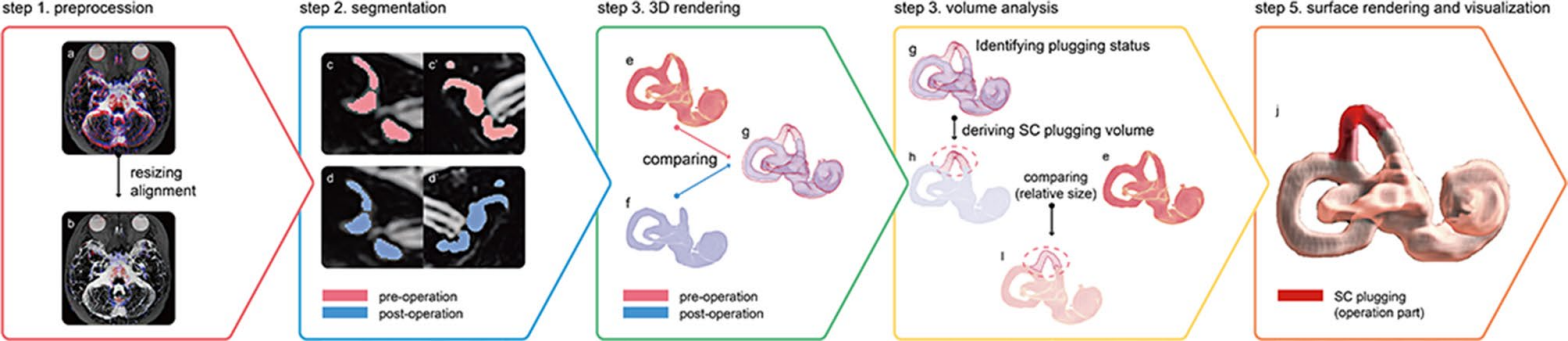
SC volume analysis workflow. (**a**) Pre- and post-operative magnetic resonance imaging (MRI) scans are overlaid (differences marked in blue and red). (**b**) Pre- and post-operative MRI scans after resizing and alignment. Segmentation of the vestibular system based on (**c**) pre- and (**d**) post-operative data. Volume rendering of the segmentation result based on (**e**) pre- and (**f**) post-operative data. (**g**) Overlay of (**f**) onto (**e**). (**h**) Manual removal of voxels outside the filling defect region. (**i**) Volume difference representing the filling defect after surgery (marked in blue). (**j**) Three-dimensional visualization based on surface rendering using direct volume rendering. The red architecture indicates the volume difference between preoperative and postoperative data and represents the filling defect after surgical plugging. This cartoon for the SC volume analysis workflow was illustrated by Adobe illustrator 2020 (https://www.adobe.com/).

Images from the seven patients were used in this study. Pre- and post-operative T2-weighted images were available for four patients where in the other three patients only postoperative images were measured. The pre- and post-operative MRI scans differed in size and orientation because of imaging settings and patient posture. Image resizing and alignment processing were applied to pre- and post-operative images. First, one image was resized using interpolation to ensure that its voxel size matched that of the other image. Next, one image was aligned to the other by means of rigid image registration (Fig. 2a,b).

### Segmentation

Because of changes in imaging conditions over time, MRI scans from the same patient taken pre and post the surgery may have different signal intensity profiles^14^. Therefore, using the same intensity threshold for pre- and post-operative images does not guarantee correct segmentation of the labyrinth fluid signal. To address this issue, Otsu’s automatic thresholding algorithm was used in this study^15^. Otsu’s automatic thresholding algorithm finds a single intensity threshold that separate pixels into two classes, foreground and background. This threshold is determined by minimizing intra-class intensity variance. To apply the appropriate threshold value, the location of the vestibular structure was identified in pre- and post-operative images. Then, a region containing the structure was defined and thresholding was applied within the region (Fig. 2c,d). In this process, we developed the in-house software for research purpose. The software is built using C++ and OpenGL API.

### Measurement of volume changes

In this study, we developed a novel 3D reconstruction-based method that combines manual segmentation of the labyrinth and an automatic thresholding algorithm to visualize the surgical plugging status of a dehiscent SC. In this process, assessment of the 3D volume of the SC plugging and its application to interpret the clinical results are indispensable. Given the quality discrepancy between preoperative and postoperative images, such as image quality and resolution due to the difference in imaging acquisition time, a direct comparison between pre- and post-operative images was considered inaccurate. To address this issue, volume changes due to a filling defect were determined as its relative size with referent to the preoperative volume, rather than absolute volume changes. More specifically, the 3D segmented volume from the postoperative MRI scan was overlapped with that from the preoperative MRI scan (Fig. 2e–g) to identify the surgical plugging status, in terms of the filling-defect topology. However, overlapping the two segmented volumes demonstrated a difference on the surface of the entire labyrinth fluid signal because the two surfaces, preoperatively and postoperatively, generally did not match perfectly. From this perspective, by manually removing the voxels outside the filling-defect region (Fig. 2g), a 3D structure representing the plugging status can be derived (Fig. 2h), and its relative size with respect to the preoperative volume was calculated (Fig. 2i). For the remaining three patients without preoperative images, the non-operated ear region was flipped and aligned to the opposite ear, and the difference was measured using a process similar to that described above.

### Visualization

For a better understanding of postoperative 3D structural changes in the semicircular canals, a direct volume rendering method was used^16^. To inspect the semicircular canal structure alone, the segmentation result was used as a binary mask to select the canal region in the image. Direct volume rendering mapped data values to color values (RGBA) using a transfer function and alpha compositing by ray casting (Fig. 2j). Because the user can assign an arbitrary transparency (alpha) to each voxel, the surface and internal structures can be visualized by means of a see-through view. The transfer function used for rendering was selected by a visualization expert based on the histogram of the labyrinth fluid signal, such that each structure could be visually distinguished.

### Audiological evaluation

The air-conduction hearing thresholds for seven octave frequencies (0.25, 0.5, 1, 2, 3, 4, and 8 kHz) and bone-conduction hearing thresholds for six frequencies (0.25, 0.5, 1, 2, 3, and 4 kHz) were recorded using pure-tone audiometry with standard audiometric testing procedures (ANSI, 1978, New York) in a soundproof booth. The technique of masking is necessary to isolate the test ear and ensure the results obtained are true thresholds of the test ear. In this study, masking was also used in bone-conduction testing when an unmasked air–bone gap is observed on the test ear. Serial audiograms were used to retrieve the hearing threshold at all frequencies on all participants preoperatively and 3 months after surgery.

### Electrophysiological evaluation

In this study, electrophysiological testing was evaluated on all participants preoperatively and 3 months after surgery. The threshold of cervical vestibular evoked myogenic potential (cVEMP) was determined by lowering the sound stimulus from the 93-dB normalized hearing level in 5-dB decrements. Alternating tone bursts (500 Hz; rate, 2.1/s; rise–fall time, 2 ms; plateau time, 3 ms; 128 repetitions; Navigation Pro; Biologic Systems, Mundelein, IL, USA) were provided to each ear as described previously^2^. The analysis time for each stimulus was 50 ms. Responses elicited by up to 80 stimuli were averaged for each test. In addition, the amplitudes from baseline to summating potential and action potential peaks were elicited using extratympanic electrocochleography with a commercial acoustic evoked potential unit (Navigation Pro ver. 7.0.0; Biologic Systems)^3^. Stimuli consisting of alternating 100-ms-duration polarity clicks (band-pass filtered, 10 Hz–1500 Hz) were presented at an intensity of the 90-dB normalized hearing level. Two replicates of averaged responses elicited by 1000–1500 clicks at 7.1 clicks/s were obtained.

### Video head impulse test: follow-up protocol and parameters

As described in our previous study^2^, all patients underwent serial video head impulse tests for the acquisition and analysis of VOR gain during impulse stimulation (ICS Impulse, GN Otometrics, Taastrup, Denmark) before surgery, immediately after surgery (i.e., within 1 week), and at 1, 3, and 6 months after surgery. The normal VOR gains were defined as > 0.8 for lateral canals and > 0.7 for vertical canals^2^.

### Statistical analysis

**Figure 3:**
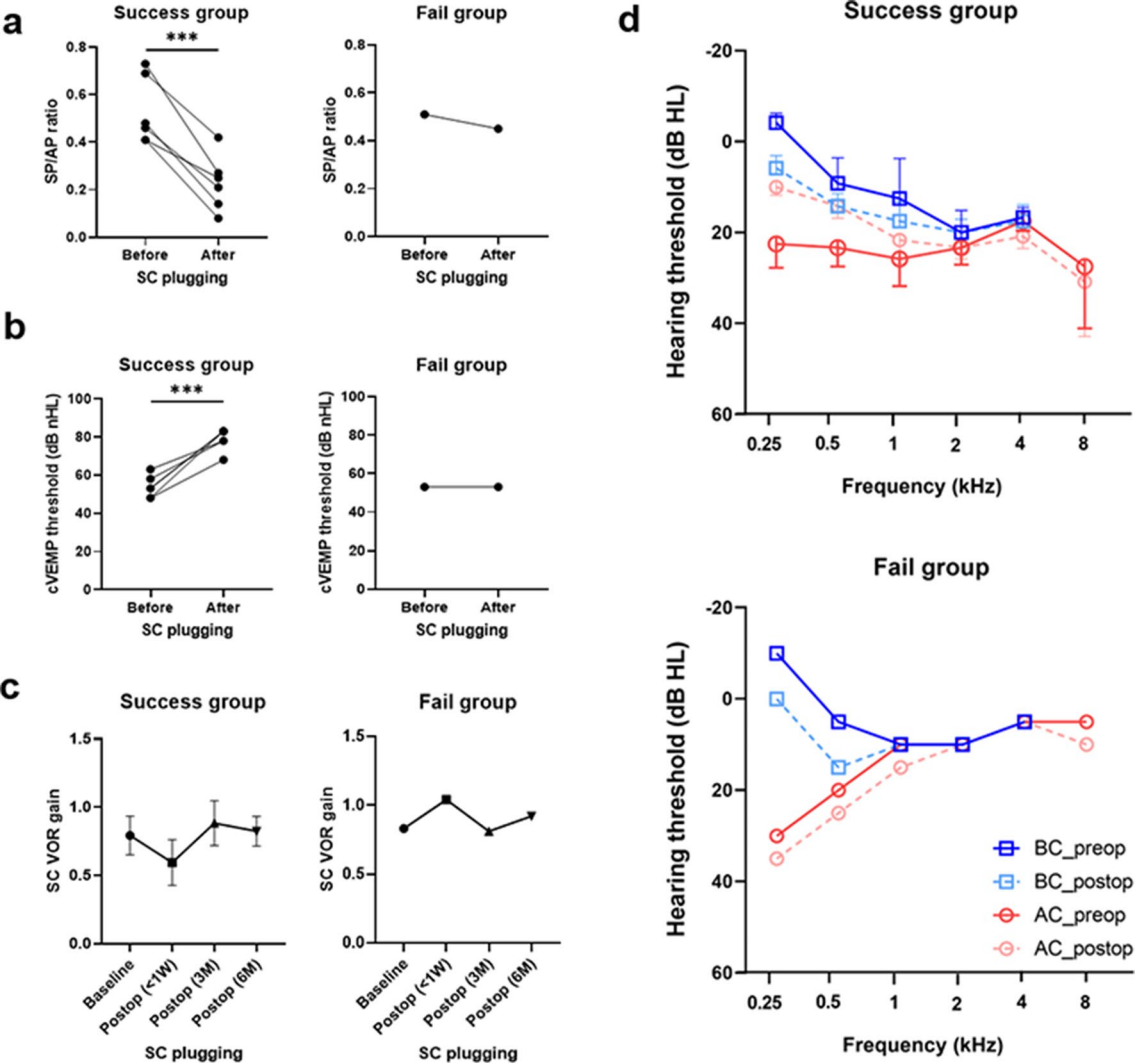
Changes in electrophysiological, vestibular, and audiological functions following SC plugging between the success and failure groups. (**a**) Summating potential/action potential ratios before and after surgery for all patients. Preoperative and postoperative gain values for the same patient are connected by a line. At 3 months after surgery, the mean summating potential/action potential ratio for operated ears had increased significantly in the success group (P < 0.001, paired t-test) but not in the failure group. ***P < 0.001. (**b**) Cervical vestibular evoked myogenic potential (cVEMP) threshold before and after surgery for all patients. At 3 months after surgery, the mean cVEMP threshold (dB nHL) in operated ears increased in the success group (P < 0.001, paired t-test) but not in the failure group. (**c**) Natural course of the mean vestibulo-ocular reflex (VOR) gain in the SC over 1 year (before and after surgical plugging). ***P < 0.001. In the success group, the mean VOR gain for plugged SCs decreased immediately (within 1 week after surgery) but improved over time to reach the preoperative value. By contrast, a fluctuating pattern for the VOR gain in the plugged SC over time was observed in the failure group. Data are presented as means ± standard errors of the mean. (**d**) Preoperative and postoperative bone conduction and air conduction thresholds are shown for all tested frequencies. In the success group, air-bone gaps (ABGs) were significantly reduced after SC plugging, particularly at low frequencies (250 and 500 Hz), due to elevated bone conduction thresholds and reduced air conduction thresholds at low frequencies (250 and 500 Hz), indicating significant improvements (P < 0.001, paired t-test). By contrast, the ABGs remained unchanged in the failure group.

The data are presented as means ± standard errors of the mean. All statistical analyses were performed using R Statistical Software (R version 3.5.2: Foundation for Statistical Computing, Vienna, Austria) and RStudio (RStudio-1.2.5042: Integrated Development for R. RStudio, Inc., Boston, MA URL http://www.rstudio.com/). Then, all the analyses (Fig. 3) were validated and illustrated using the GraphPad Prism version 9.0.0 for Windows, GraphPad Software, San Diego, California USA (www.graphpad.com). In this study, paired t tests were used as appropriate to compare cVEMP thresholds, SP/AP ratios, and pure-tone audiometry before and after canal plugging since the variables were normally distributed. All statistical tests were two-tailed, and P < 0.05 was considered to indicate statistical significance. The inter-class correlation was performed to determine the consistency of average measures among the rates via Kappa statistic. The inter-class correlation coefficient for labyrinth fluid signal and SC plugging volume was determined based on the results. All statistical tests were two-tailed, and P < 0.05 was considered to indicate statistical significance.

## Results

### Demographic and clinical characteristics

The demographic and clinical characteristics of the seven patients enrolled in this study are summarized in Table 1. Five patients had unilateral SCD, whereas two patients had bilateral SCD. The two patients with bilateral SCD underwent canal plugging only for the more symptomatic ear via the middle fossa approach. The mean age was 44.4 ± 3.58 years (range, 26–56 years), and four patients were women. Most patients had SCD at the arcuate eminence, whereas one patient had SCD at the level of the SPS near the common crus.

**Table 1:** Demographics and clinical characteristics of patients with superior semicircular canal dehiscence.

Case no.	Sex/age	Operation (approach)	Location	FU period^a^ (months)	Autophony		Ear fullness		Subjective hearing loss		Dizziness		Tullio/Hennebert		Pulsating tinnitus	
					Pre	Post	Pre	Post	Pre	Post	Pre	Post	Pre	Post	Pre	Post
1	F/40	R) plugging (MFA)	AE	17	●*	NP	●	NP	NP	NP	●	NP	NP	NP	●	○
2	F/50	L) plugging (MFA)	SPS encasing	12	●*	○	●	NP	●	○	●	NP	NP	NP	●	○
3	F/47	R) plugging (MFA)	AE	7	●*	NP	●	NP	●	NP	NP	NP	NP	NP	●	NP
4	M/56	L) plugging (MFA)	AE	10	●*	NP	NP	NP	NP	NP	●	NP	NP	NP	●	NP
5	M/47	L) plugging (MFA)	AE	36	●*	NP	●	NP	NP	NP	●	NP	●	NP	●	NP
6	M/26	L) plugging (MFA)	AE	43	●	NP	●	NP	NP	NP	●*	○	●	NP	●	NP
7	F/45	R) plugging (MFA)	AE	31	●	NP	NP	NP	NP	NP	●*	○	●	NP	●	○

*M* male, *F* female, *R* right, *L* left, *B* bilateral, *MFA* middle fossa approach, *AE* arcuate eminence, *SPS* superior petrosal sinus, *FU* follow up, *NP* not present, ● present, ○ improved but remained.

*Chief complaint.

^a^Note that refers to period of follow-up from the surgery to the present. The status if postoperative symptoms after surgery is based on the present.

### Subjective symptoms before and after SC plugging

As summarized in Table 1, there was an average of four preoperative cochleovestibular symptoms per patient (range, 3–5). Autophony and pulsatile tinnitus were the most common symptoms (100.0% of patients), followed by dizziness (85.7%), ear fullness (71.4%), and Tullio phenomenon/Hennebert signs (42.9%), and subjective hearing loss (28.6%). While the Tullio phenomenon refers to sound-induced vertigo, nystagmus, or both, Hennebert sign is pressure-induced vertigo, nystagmus, or both, elicited by insufflation of the external auditory canal. During the follow-up period (median: 17 months, range: 7–43 months), subjective symptoms were absent or markedly relieved in most patients. However, one patient (Case No. 2) experienced persistent residual symptoms, including autophony, subjective hearing loss, and pulsatile tinnitus. High-resolution temporal bone CT of the patient revealed that left SPS running along the petrosal ridge abuts SC instead of the middle cranial fossa, exhibiting the bony dehiscence of the SC by the SPS, in which case the location of SCD is closer to the common crus than the ampulla. Furthermore, the pattern of left-side 500 Hz tone-induced nystagmus in that patient was mainly torsional with few vertical components, presumably due to co-stimulation of the superior and posterior canals^17^. Accordingly, our cohort was divided into two subgroups depending on SC-plugging outcome: success (n = 6) versus failure (n = 1).

### Vestibular function before and after SC plugging

The mean summating potential/action potential ratio in operated ears decreased significantly from 0.50 ± 0.04 (preoperatively) to 0.30 ± 0.02 (postoperatively) (95% confidence interval =  − 0.28 to − 0.11, P < 0.001 by paired t-test) (Fig. 3a). Consistent with this result, SC plugging significantly enhanced the mean cVEMP threshold (dB nHL) in the operated ears of all patients in the success group, compared with the baseline (from 53.83 ± 2.39 (preoperatively) to 78.83 ± 2.39 (postoperatively), 95% confidence interval = 14.1–29.5, P < 0.001 by paired t-test) (Fig. 3b). However, the electrophysiological results of the one patient in the failure group were nearly equivalent before and after surgery, without any improvements. In addition, the VOR gain for the plugged SC immediately deteriorated (within 1 week) postoperatively but was subsequently restored in most patients. Notably, one patient had persistent residual symptoms (Fig. 3c), such that the VOR gain in the plugged SC exhibited a fluctuating pattern over time, eventually resulting in a marked reduction in VOR gain at the last evaluation. The VOR gain for the other semicircular canals did not differ significantly between time points, with nearly equivalent values observed for each canal over a 1-year period.

### Audiological characteristics before and after SC plugging

The mean air-bone gaps (ABGs) across 0.25, 0.5, and 1 kHz decreased significantly from 20.0 ± 2.6 dB at baseline to 6.5 ± 2.4 dB postoperatively (95% confidence interval =  − 20.8 to − 6.2, P = 0.002 by paired t-test) in the success group (Fig. 3d). Specifically, the bone conduction thresholds at 0.25 and 0.5 kHz for those patients increased from − 3.6 ± 1.5 dB at baseline to 5.9 ± 2.2 dB postoperatively (P < 0.001 by paired t-test) and 5.5 ± 3.1 dB at baseline to 12.7 ± 2.1 dB postoperatively (P = 0.009 by paired t-test), respectively. Conversely, the mean ABGs across 0.25, 0.5, and 1 kHz for one patient with residual symptoms (i.e., failure group) remained unchanged despite SC plugging.

### Volumetric measurements before and after SC plugging

**Figure 4:**
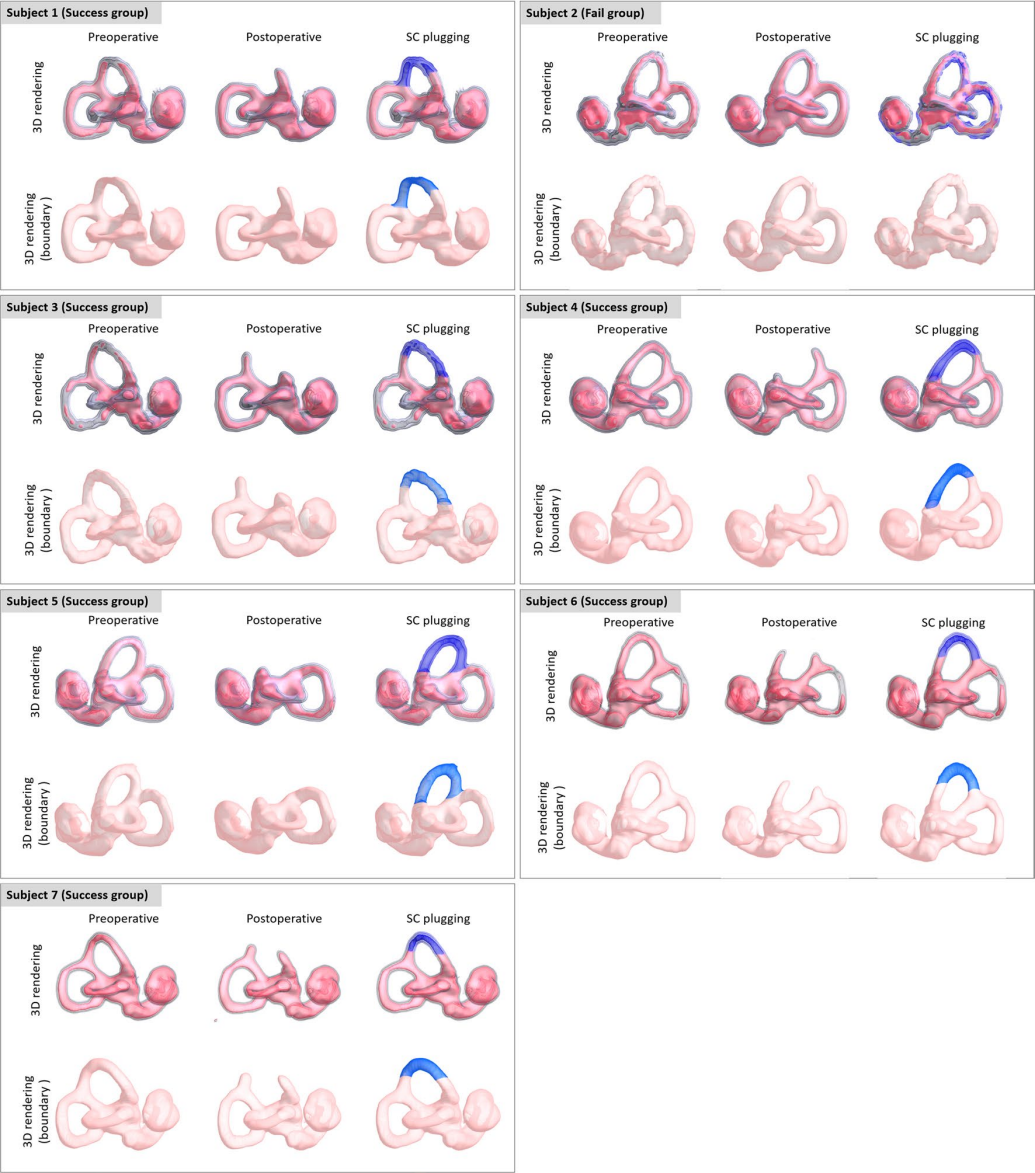
Individual 3D-based visualization of SC-plugging status using surface and direct volume rendering. The blue architecture indicates the volume difference between preoperative and postoperative data and represents the filling defect after surgical plugging. The images in this figure were created using a C++ and OpenGL-based program by our co-authors. Therefore, no external entity was used and no citation is required.

Individual data regarding the status and extent of SC plugging in all patients are depicted in Fig. 4. Volumetric reconstruction images were available for all patients and revealed filling defects at the sites of SC plugging, which corresponded with complete obliteration of the patent SC visible on preoperative imaging. Variable extents of filling defects were observed at the sites of SC plugging, in addition to variations in the positional relationship of the defect with the ampulla and common crus. The mean 3D volumetric change after SC plugging (i.e., extent of SC plugging) was 5.20 ± 1.07% (range, 0–9.27%) of the total volume of the preoperative labyrinth fluid signal. Specifically, the inter-class correlation was excellent regarding 3D volumetric changes (Table 2). Overall, patients in the success group exhibited markedly reduced individual volume changes following SC plugging, whereas the volume remained unchanged (indicating patent SC) in the failure group. These results clearly indicate that the success or failure of SC plugging was related to 3D volume changes in the labyrinth fluid signal.

**Table 2:** Inter-class correlation in the evaluation of the qualitative analysis.

	Inter-class correlation coefficient (*Kappa*)^a^	P-value
Membranous labyrinth volume (baseline)	1.000	< 0.001
SC plugging volume (operation)	0.999	< 0.001

^a^Note that inter-class correlation was performed to determine the consistency of average measures among the rates via Kappa statistic. The consistency level was defined as follows: *Kappa* < 0.6, poor consistency; 0.4 ≤ Kappa < 0.79, general consistency; and Kappa ≥ 0.80, good consistency; SC, superior semicircular canal.

## Discussion

To the best of our knowledge, this is the first study to evaluate the extent and status of SC plugging using a 3D reconstruction algorithm derived from conventional MRI. This novel algorithm allows individualized visualization of SCD, while enabling accurate volumetric assessment of the extent of any filling defect in the peri-lymphatic fluid space. Although this was a preliminary study, the significant correlation between volumetric change from surgical plugging and objective measurement of dehiscence volume strongly indicates that this novel method is extremely reliable. This approach may be broadly applicable following validation of the postoperative status of SC plugging and can be part of an additional treatment strategy for patients with residual symptoms.

Multiple studies have demonstrated that sufficient canal plugging provides relief from clinical symptoms. However, some patients remain symptomatic despite canal plugging, presumably because of incomplete surgical plugging^8, 18^. In this study, one patient (failure group) remained symptomatic despite SC plugging through the middle fossa approach and exhibited abnormal functional results, implying incomplete plugging of the operated SC. The SCD was caused by a deep SPS groove, consistent with findings from a previous temporal bone study, which confirmed that the SPS is the second-most proximal structure to the SC (after the middle cranial fossa floor)^19^. A deep SPS groove may cause SCD near the common crus^1^, likely precluding complete plugging at the site of dehiscence due to difficulty in separating the SPS attachment from the dehiscence or a relatively smaller dehiscence, compared with the SCD, at the arcuate eminence. Subsequently, a clear contrast in terms of 3D reconstruction-based volumetric changes was observed between the success and failure groups. These data suggest that information concerning pre- and post-operative volumetric changes may help in determining patient prognosis. The clinical significance may be more apparent when the assessment is blinded to specific objective measures, such as cVEMP threshold and summating potential/action potential ratio, indicative of third mobile window effects. Although the dehiscent SC volume might serve as a potential predictor of symptom outcomes after surgical plugging^8, 9^, no study has evaluated the causality of volumetric changes after SC plugging with regard to subjective and objective improvements.

The novel 3D reconstruction technique using a post hoc MRI processing algorithm can be used to identify positional relationships relative to important landmarks (e.g., ampulla or common crus) within the semicircular canals. For instance, each ampulla contains a cupula on top of vestibular hair cells consisting of a crista ampullaris. Cupula deflection opposing the direction of head movement transduces corresponding electrical signals in the vestibular hair cells, which are transmitted to the brain through the vestibular nerve^20^. Recently, we reported that successful plugging of a dehiscent SC was closely associated with a transient disturbance in labyrinthine activity exclusively involving the plugged SC^2^. This finding, together with findings from animal studies, may be mainly attributable to peripheral recovery processes and residual sensitivity of the plugged SC to angular head acceleration^21, 22^, suggesting the importance of ampulla preservation during SC plugging. A recent study demonstrated that canal plugging involving the superior ampulla caused acute cochleovestibular deficits (i.e., postoperative labyrinthitis) with labyrinthine enhancement involving the entire labyrinth as observed in post-contrast 3D fluid-attenuated inversion recovery images^8^. Similarly, Charpiot et al. suggested that canal plugging, at least 3 mm from the ampulla, can reduce the risk of acute cochleovestibular syndrome^23^. Collectively, these findings suggest that intuitive and finely tuned visualization of plugging by a 3D reconstruction algorithm, irrespective of superior ampulla involvement, can likely be used to predict transient or permanent vestibular function and the likelihood of postoperative cochleovestibular complications^24^. These results may have clinical implications concerning timely and tailored vestibular rehabilitation therapy for patients with residual dizziness.

This study had some limitations that should be addressed in future studies. First, it included a small number of patients, which may have weakened the clinical implications of the results and statistical power. Therefore, large-scale studies are warranted. Second, it remains unclear whether the clinical effectiveness of our 3D reconstruction algorithm is superior to that of conventional techniques, such as maximum intensity projection reconstruction, in terms of evaluating SC-plugging status. To the best of our knowledge, no study has implemented such meticulous visualization of SC plugging with volume computation based on 3D reconstruction. Third, according to Chemtob et al., co-registration of CT/MRI will enable accurate and routine localization of residual defects in the SC following plugging^18^. In that context, there is a need to maximally adjust complete overlap without residual defects (e.g., using CT/MRI co-registration), which should facilitate successful clinical outcomes. Lastly, the structure of the labyrinth, including the semicircular canals, is relatively small and irregular; therefore, the resolution of clinical MRI is insufficient for consistent segmentation. Although good inter-class correlation between the two observers was observed in this study, the use of data-driven approaches (e.g., deep learning) could further automate the process and improve the accuracy and robustness of segmentation. This will eventually enhance the clinical significance of 3D reconstruction algorithm-based volumetry.

In conclusion, this novel method of evaluating dehiscence size based on 3D reconstruction is expected to enable elaborate visualization of SC-plugging status with volumetric assessment. Furthermore, the results were significantly correlated with postoperative clinical outcomes. In this era of precision medicine, the current study contributes to establishment of a future “new normal” protocol for routine 3D reconstruction-based volumetry, which will guide treatment for patients with residual symptoms and abnormal functional test results. The current technology will facilitate diagnosis of various diseases and exploration of morphometric changes in multiple target organs.

## Supplementary Information

Supplementary Table 1.
