# Supplementary material for: Quantitative three-dimensional image analysis of the superior canal after surgical plugging to treat superior semicircular canal dehiscence
Source: Sci Rep. 2021 Aug 9;11:16112. doi: 10.1038/s41598-021-95063-x (PMC8352905; doi:10.1038/s41598-021-95063-x)
Supplement: Supplementary file 1 — Supplementary Table 1. [file 41598_2021_95063_MOESM1_ESM.pdf]

Quantitative three-dimensional image analysis of the superior canal after surgical plugging to treat superior semicircular canal dehiscence

Sang-Yeon Lee, Yein Lee, Jun Young Choi, Yun Jung Bae, MinJu Kim, Jae-Jin Song, Byung Yoon Choi, Won-Ki Jeong, Ja-Won Koo

Supplementary Table 1. Profile of MR image resolution on each patient included in this study

**Case No.**		original size (voxel)	original resolution ($mm3$ per voxel)	resized size (voxel)	resized resolution (mm^3 per voxel)	**volume per voxel (mm^3)**
**1**	Preoperative	512x512x70	0.312x0.312x0.7	512x512x157	0.312^3	0.030371328
	postoperative	480x480x140	0.333x0.333x0.35	512x512x157	0.312^3	0.030371328
**2**	Preoperative	528x528x70	0.284x0.284x0.7	528x528x173	0.284^3	0.022906304
	postoperative	480x480x140	0.333x0.333x0.35	563x563x173	0.284^3	0.022906304
**3**	Preoperative	528x528x70	0.284x0.284x0.7	528x528x173	0.284^3	0.022906304
	postoperative	480x480x140	0.333x0.333x0.35	563x563x173	0.284^3	0.022906304
**5**	Preoperative	480x480x59	0.333x0.333x0.35	480x480x62	0.333^3	0.036926037
	postoperative	480x480x140	0.333x0.333x0.35	480x480x147	0.333^3	0.036926037
**4**	Preoperative	Not available	Not available	Not available	Not available	
	postoperative	480x480x140	0.333x0.333x0.35	480x480x147	0.333^3	0.036926037
**6**	Preoperative	Not available	Not available	Not available	Not available	
	postoperative	480x480x140	0.333x0.333x0.35	480x480x147	0.333^3	0.036926037
**7**	Preoperative	Not available	Not available	Not available	Not available	
	postoperative	480x480x140	0.333x0.333x0.35	480x480x147	0.333^3	0.036926037
